# Standardizing Ankle Position During the Active Straight Leg Raise: Effects on Lateral Abdominal Muscle Thickness and Pressure Biofeedback in Healthy Young Adults

**DOI:** 10.3390/jfmk11030337

**Published:** 2026-08-27

**Authors:** Yinghong Liu, Xiaoliang Qiu, Dae-Sung Park

**Affiliations:** Department of Physical Therapy, Konyang University, 158 Gwanjeodong-ro, Seo-gu, Daejeon 35365, Republic of Korea

**Keywords:** ASLR, clinical assessment, protocol standardization, physical therapy, RUSI, pressure biofeedback, lumbopelvic control

## Abstract

**Background and Objectives:** Ankle instructions during the active straight leg raise (ASLR) are not consistently standardized and may alter lumbopelvic measurements. This study tested whether a relaxed ankle, maximal active dorsiflexion (DF), and maximal active plantarflexion (PF) changed lateral abdominal muscle thickness and pressure biofeedback unit (PBU) responses during a standardized supine ASLR performed at 30° of hip flexion. **Methods:** Thirty-three healthy adults completed three trials per condition according to a pre-specified schedule containing all six possible condition orders. B-mode rehabilitative ultrasound imaging (RUSI) measured transversus abdominis (TrA), internal oblique (IO), and external oblique (EO) thickness at rest and during ASLR. Three end-expiratory images were acquired for each participant, condition, and state; each image was measured three times, and the three image means were averaged. Rest-to-ASLR changes yielded 99 participant-condition values. Pressure biofeedback unit (PBU) pressure was recorded at four prespecified events and analyzed at the trial level. **Results:** Across participant-condition means, TrA thickness was 2.44 ± 0.59 mm at rest and 2.66 ± 0.70 mm during ASLR; the largest observed mean was 4.46 mm under the study task, not a physiological maximum. DF increased the change in transversus abdominis thickness (ΔTrA) versus the relaxed ankle by 0.172 mm (95% CI, 0.077–0.267; *p* < 0.001) and total lateral abdominal wall change by 0.672 mm (95% CI, 0.413–0.931; *p* < 0.001). PF increased signed image-moment pressure deviation by 1.505 mmHg (95% CI, 0.985–2.025; *p* < 0.001). **Conclusions:** Ankle-position instructions changed ultrasound and PBU outcomes during ASLR. Standardizing ankle positioning instructions and image aggregation may improve measurement reproducibility.

## 1. Introduction

Effective neuromuscular control of the trunk coordinates abdominal muscle activity with pelvic and lower-limb movement, limits unwanted lumbopelvic motion, and supports efficient load transfer during functional tasks [1,2,3]. When this control is impaired, individuals may adopt compensatory movements or altered pressure strategies, which can complicate rehabilitation assessment and reduce the reproducibility of functional testing.

Against this background, the active straight leg raise (ASLR) is a useful clinical and experimental task because it challenges trunk control during unilateral limb movement against gravity while allowing hip flexion angle to be standardized. The ASLR also permits concurrent assessment of abdominal muscle morphology and lumbopelvic pressure [1,2,3,4,5,6,7,8]. Supine leg-raising tasks are common in rehabilitation because they combine lower-limb movement with a demand for lumbopelvic control. The ASLR test is used to examine interactions among lower-limb loading, pelvic control, and abdominal wall function in healthy and symptomatic populations [1,2,3]. In clinical exercise and physical therapy, the task may be performed with the ankle relaxed, in dorsiflexion (DF), or in plantarflexion (PF). If these distal cues alter the measured response, inconsistent cueing could reduce the comparability of repeated assessments and the transferability of research protocols.

Rehabilitative ultrasound imaging (RUSI) provides a noninvasive method for quantifying transversus abdominis (TrA), internal oblique (IO), and external oblique (EO) thickness at rest and during movement [9,10,11,12,13]. Thickness change reflects a morphological response associated with muscle behavior but is not a direct surrogate for electromyographic activity, because the relationship between ultrasound thickness and electromyographic amplitude varies across abdominal muscles and tasks [14,15]. Active DF and PF could influence the ASLR through ankle-muscle contraction, posterior-chain tension, neurodynamic effects, or lower limb-trunk coordination. DF during the ASLR alters tibial nerve excursion and strain [16,17], and a preliminary randomized study found a greater TrA response when abdominal drawing-in was combined with DF [18]. However, relaxed, DF, and PF ankle positions have not been compared within the same standardized ASLR protocol.

Muscle thickness alone does not characterize lumbopelvic control during the ASLR. Pressure changes beneath the lumbopelvic region can be assessed with a pressure biofeedback unit (PBU). Although PBU measurements are used to assess and train lumbopelvic control and to provide feedback during abdominal drawing-in tasks [4,5,6,7], they do not directly measure TrA activation or global core stability. Examining ultrasound and PBU outcomes together may therefore clarify whether morphological and pressure responses follow similar patterns across ankle positions.

This study compared a relaxed ankle, maximal active DF, and maximal active PF during a standardized supine ASLR performed at 30° of hip flexion in healthy adults. The primary outcome was the change in transversus abdominis thickness (ΔTrA), defined as active straight leg raise (ASLR) thickness minus rest thickness. Secondary outcomes included changes in internal oblique thickness (ΔIO) and external oblique thickness (ΔEO), change in total lateral abdominal wall thickness, the relative contribution of TrA to total wall thickness, and PBU pressure indices. By identifying a potential source of measurement variation, the study aimed to inform more reproducible ASLR assessment procedures in rehabilitation practice and research. We hypothesized that ankle positioning cues would alter these measurements despite a constant posture, limb, and hip flexion angle.

## 2. Materials and Methods

### 2.1. Study Design and Participants

This exploratory, single-group study used a within-participant repeated-measures design. The study protocol was approved by the Institutional Review Board of Konyang University (Approval No. KYU 2026-05-066-001). All participants provided written informed consent before testing.

An a priori power analysis was performed using G*Power version 3.1.9.7 (Heinrich Heine University Düsseldorf, Düsseldorf, Germany) for a within-participant repeated-measures design. Assuming a medium effect (f = 0.25), an alpha level of 0.05, a power of 0.80, three repeated condition measurements, a correlation of 0.55 among repeated measures, and a nonsphericity correction of 0.75, the minimum required sample size was 30. This target was intended for an exploratory within-participant comparison rather than definitive inferences regarding clinical effectiveness.

Participants were recruited through notices posted on institutional bulletin boards and online recruitment channels. Forty healthy adults aged 18–40 years responded to the recruitment notices and were assessed for eligibility. Eligible volunteers provided written informed consent before testing. The inclusion criteria included no low back pain during the previous 3 months, no current pain in the hip, abdomen, or lower limb, and the ability to independently raise one lower limb to at least 30° without assistance. The exclusion criteria were current musculoskeletal pain or neurologic disease, including lumbar disc herniation; abdominal, lumbar, pelvic, or lower-limb surgery within the previous 6 months; long-term specialized core training, including employment as a yoga or Pilates instructor; pregnancy or less than 6 months postpartum; severe ankle deformity or restricted ankle mobility; or ultrasound images in which the muscle borders could not be clearly identified. Initially, 40 healthy adults aged 18–40 years responded and were assessed for eligibility. Seven were subsequently excluded because blurred ultrasound images prevented reliable identification of fascial borders across the repeated-measures dataset. The final analytical sample comprised 33 participants (17 male and 16 female; mean age, 24.70 ± 1.74 years).

The three ankle conditions were administered according to a pre-specified schedule containing all six possible condition orders. One condition sequence was assigned to each of the 40 recruited participants before testing. After seven exclusions, all six sequences remained represented, with 4–7 participants per sequence. The schedule file preserved the assigned sequences but not the software, randomization algorithm, random seed, or allocation concealment details. Complete condition-order information was available for all 33 analyzed participants, who contributed 297 trial records. Because sequence and period were not included as fixed effects in the primary models, potential fatigue, learning, period, and carryover effects could not be excluded.

### 2.2. Experimental Task and Ankle Conditions

Participants were positioned supine with the head in a neutral position, the arms alongside the body, and both lower limbs fully extended. They actively raised their dominant lower limb to 30° of hip flexion while maintaining knee extension. Hip flexion angle was verified using a digital goniometer (2-in-1 Digital Angle Ruler, BODY ANGLE; BODY BUDDY, marketed by CodeCrunch, Anyang, Republic of Korea; resolution, 0.05°; stated accuracy, 0.5°). The raised limb was held at 30° of hip flexion for 5 s during each ASLR trial. Compensatory movements, such as trunk flexion or posterior pelvic tilt, were strictly prohibited. Ultrasound images were captured at the end of expiration. To minimize muscular fatigue, a 2-min rest interval was enforced between trials [4,7].

During the relaxed-ankle condition, participants were instructed not to actively perform DF or PF; the natural resting angle of the ankle was established as the participant-specific 0° baseline. Subsequent DF and PF excursions were calculated relative to this reference. In the active conditions, participants moved the ankle to the maximal achievable DF or PF and maintained this position throughout the ASLR. Consequently, these conditions coupled specific joint positioning with active ankle muscle contractions.

### 2.3. Ultrasound and PBU Measurements

Ultrasound examinations were performed using a B-mode system equipped with a 5–12 MHz linear transducer (MySono U6; Samsung Medison Co., Ltd., Seoul, Republic of Korea). A single examiner who had completed 30 h of supervised training in abdominal musculoskeletal ultrasound acquired all images. The transducer was placed transversely on the dominant abdominal wall at the anterior axillary line, midway between the 12th rib and iliac crest. Sufficient acoustic gel was applied to minimize tissue compression by the transducer [11,12,13].

PBU pressure was assessed exclusively during the 5 s static hold phase of the ASLR. Participants actively raised their dominant lower limb over a 2 s period and then maintained it at 30° of hip flexion. Event-based readings obtained during the hold were used to calculate signed image-moment deviation, maximum absolute deviation, and root-mean-square error (RMSE); the precise definitions and equations for these indices are provided below. Consequently, PBU data were analyzed as discrete event-based values rather than as a continuous time series.

Thicknesses of the TrA, IO, and EO muscles were measured between the inner borders of the superficial and deep fascial layers. The TrA muscle fascia junction (MFJ) was identified, and thickness was quantified along a vertical line 1.5 cm medial to the junction. For each participant-condition-state cell, three end-expiratory images were acquired at rest and three during the ASLR. Offline measurements were performed using the integrated measurement software of the MySono U6 system. Each muscle was measured three times on every image; these readings were averaged to obtain one image-level value. The three image-level values were then averaged to obtain a single aggregate value per participant, condition, and state for analysis [11,13]. Before offline measurement, device-generated filenames were replaced with randomized alphanumeric codes to mask participant identity, time point, trial, ankle condition, and experimental state. Images were digitally randomized and measured in the newly generated order. During offline measurement, the examiner was blinded to the ankle condition and experimental state.

The PBU (Stabilizer Pressure Biofeedback Unit; Chattanooga Group Inc., Hixson, TN, USA) was placed horizontally beneath the lower back and lumbopelvic region and inflated to 40 mmHg [5,7]. A designated investigator manually recorded pressure when the limb reached 30°, at 3 and 5 s, and when the ultrasound image was frozen at end-expiration during the hold. The device was reset to 40 mmHg after each trial.

For event k, the pressure deviation was calculated as

d_k_ = P_k_ − P_0_

where P_k_ is the pressure recorded at event k and P_0_ = 40 mmHg is the baseline pressure. Positive values indicated pressure above baseline. The image-moment deviation was defined as

d_image_ = P_image_ − P_0_

The maximum absolute deviation (D_max_) was calculated as

D_max_ = max(|d_30°_|, |d_3_|, |d_5_|, |d_image_|)

The root-mean-square error (RMSE) was calculated from the three prespecified events as

RMSE = √[(d_30°_^2^ + d_3_^2^ + d_5_^2^)/3]

Thus, D_max_ represented the largest absolute pressure excursion across the four recording events, whereas RMSE summarized the magnitude of deviations at the three timed events. The image-moment deviation was excluded from the RMSE calculation because its timing was determined by ultrasound image acquisition rather than by a prespecified time point.

### 2.4. Outcomes and Statistical Analysis

The primary outcome was ΔTrA, defined as the TrA thickness during the ASLR minus TrA thickness at rest. Secondary ultrasound outcomes included ΔIO and ΔEO, the change in total lateral abdominal wall thickness, and the change in the relative contribution of TrA to total lateral abdominal wall thickness. PBU outcomes included the signed image-moment pressure deviation, maximum absolute pressure deviation, and pressure deviation RMSE.

Ultrasound change scores were analyzed using linear mixed-effects models with ankle condition as a fixed effect, the relaxed-ankle condition as the reference level, and participant as a random intercept. For PBU outcomes, trial-level linear mixed-effects models were fitted with ankle condition as a fixed effect, the relaxed-ankle condition as the reference level, and participant as a random intercept. All models were estimated using restricted maximum likelihood (REML). Wald estimates, 95% confidence intervals (CIs), and two-sided *p*-values are reported. The prespecified significance level (α) was 0.05. In a sensitivity analysis, paired t-tests were used to compare participant-level changes between the active and relaxed conditions, with a Holm–Bonferroni correction applied to the two planned contrasts within each outcome. The secondary ultrasound, PBU, and combined ultrasound–PBU analyses were considered exploratory. Data processing and statistical calculations were performed using Python 3.12.13 (Python Software Foundation), along with the pandas 3.0.1 and NumPy 2.3.5 libraries.

For the supplemental intra-rater reliability analysis, 50 ultrasound images were randomly selected and remeasured by the same examiner after a 15-day interval. The examiner was completely blinded to the initial measurements during the second session. Original image means were recalculated from the three recorded measurements prior to analysis. Intraclass correlation coefficients (ICCs) were calculated using a two-way mixed-effects, absolute-agreement, single-measure model [ICC(A,1)]. Bootstrapped 95% CIs were generated using 10,000 image-level resamples. The standard error of measurement (SEM) and minimal detectable change at the 95% confidence level (MDC_95_) were also calculated. Notably, these reliability statistics reflect the precision of a single image mean, whereas the primary condition-level ultrasound outcomes were derived by averaging three image means.

## 3. Results

### 3.1. Participant Characteristics and Included Observations

The analysis included 297 trial records and 594 ultrasound images from 33 participants (17 male and 16 female). All images yielded three valid thickness measurements for each muscle. For each participant, the three image means for each condition and state were averaged, yielding 198 participant-condition-state values. Subtracting the resting values from the ASLR values produced 99 participant-condition change values for the ultrasound mixed-effects models. Each participant completed three trials in each ankle condition (Table 1). The final sequence counts were 4 for relaxed-DF-PF, 5 for relaxed-PF-DF, 5 for DF-relaxed-PF, 6 for DF-PF-relaxed, 7 for PF-relaxed-DF, and 6 for PF-DF-relaxed. Consequently, the relaxed-ankle, DF, and PF conditions were administered first in 9, 11, and 13 participants; second in 12, 10, and 11 participants; and third in 12, 12, and 9 participants, respectively.

### 3.2. Ultrasound Outcomes

Mean TrA thickness was greater during ASLR than at rest across all ankle conditions (Table 2). Across the 99 participant-condition observations, TrA thickness averaged 2.44 ± 0.59 mm at rest and 2.66 ± 0.70 mm during ASLR. The largest participant-condition mean observed during the standardized task was 4.46 mm. Because no specific maximal contraction maneuver—such as one involving trunk flexion or posterior pelvic tilt—was performed, this value should not be interpreted as an absolute physiological maximum. Table 2 reports the corresponding absolute thickness summaries for each ankle condition.

**Figure 1:**
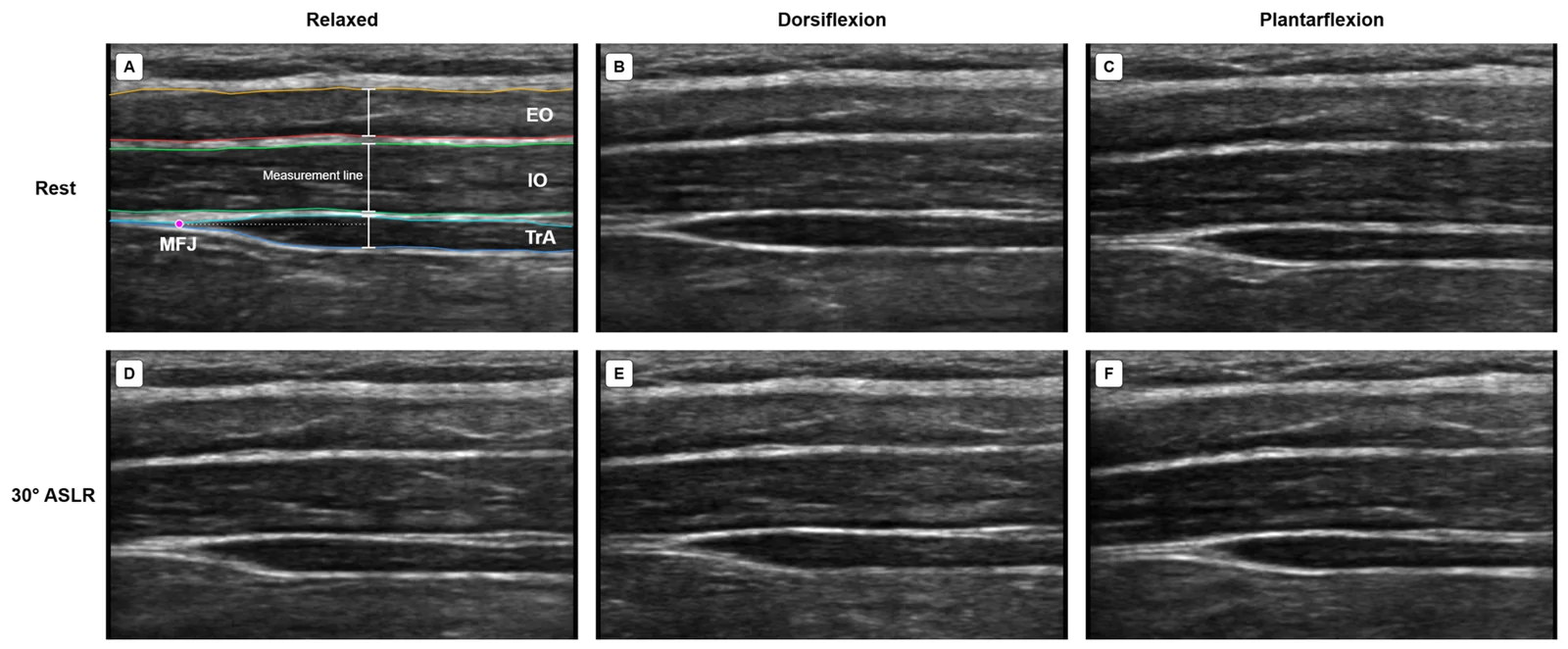
Representative B-mode ultrasound images of the lateral abdominal muscles at rest (top row) and during a 30° ASLR (bottom row) with a relaxed ankle, maximal active DF, or maximal active PF. Images were obtained from one participant using consistent settings and transducer placement. In panel (**A**), colored contours delineate the fascial borders of the EO, IO, and TrA; the magenta point marks the muscle fascia junction (MFJ), and the horizontal white dashed line indicates the 1.5 cm medial offset to the vertical measurement calipers. Panels (**B**–**F**) are unannotated to preserve anatomical visibility.

**Figure 2:**
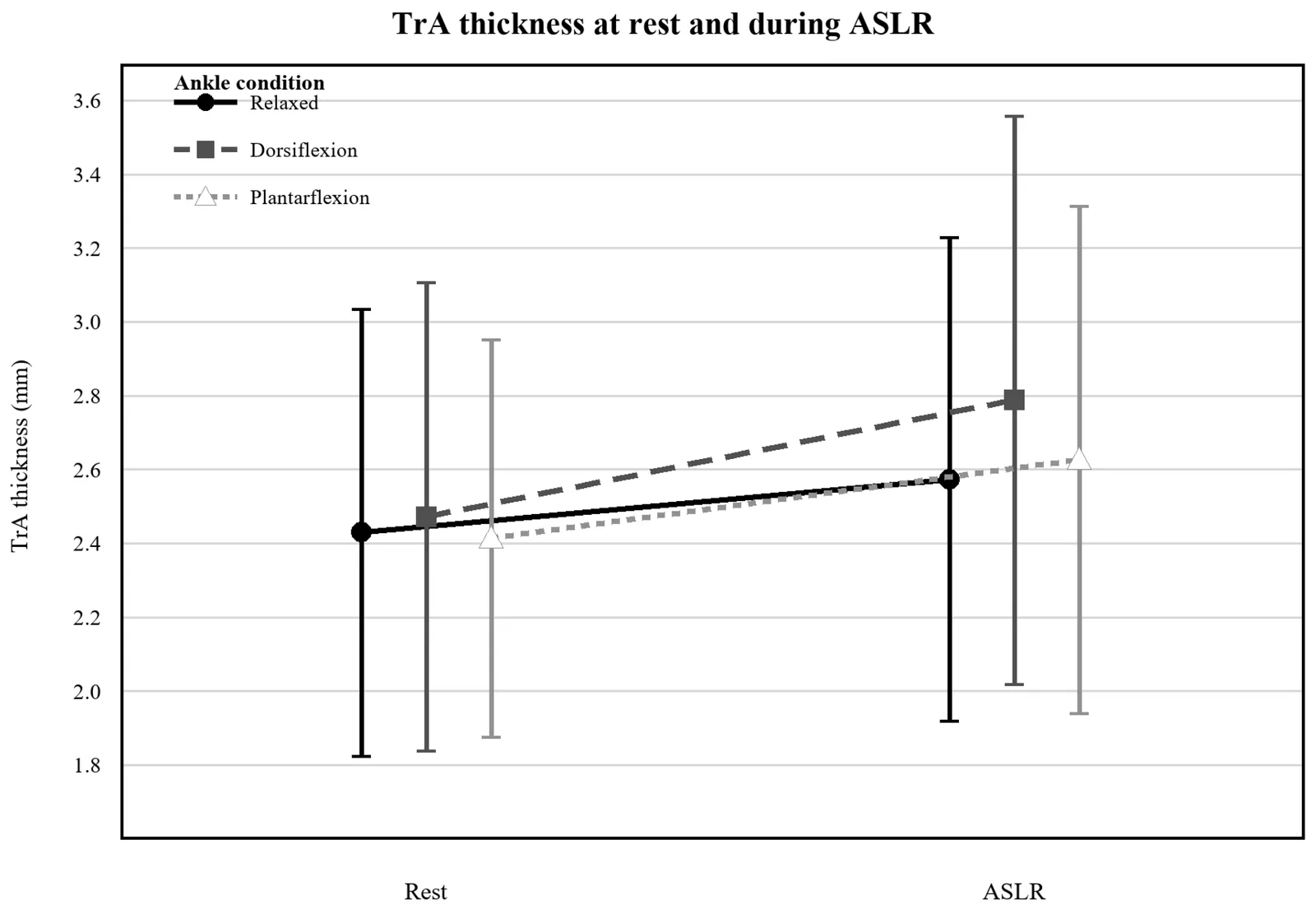
Mean TrA thickness at rest and during the ASLR across ankle conditions. Each data point represents the mean of 33 participant-level aggregate values for a specific state and condition; individual participant values were calculated by averaging three end-expiratory image measurements. Error bars indicate standard deviations (SD). The *y*-axis is truncated to 1.8–3.6 mm to improve visibility of the small within-task differences; absolute values are reported in Table 2.

In the primary linear mixed-effects model, the DF condition significantly increased ΔTrA by 0.172 mm relative to the relaxed-ankle condition (95% CI, 0.077–0.267; *p* < 0.001). To provide measurement context, this group-level estimate was smaller than the single-image intra-rater MDC95 of 0.477 mm. The planned contrast comparing the PF condition with the relaxed-ankle condition was not statistically significant (estimated difference, 0.068 mm; 95% CI, −0.027–0.163; *p* = 0.163). Figure 1 shows representative images, Figure 2 presents descriptive means, and Table 3 reports the model estimates.

**Figure 3:**
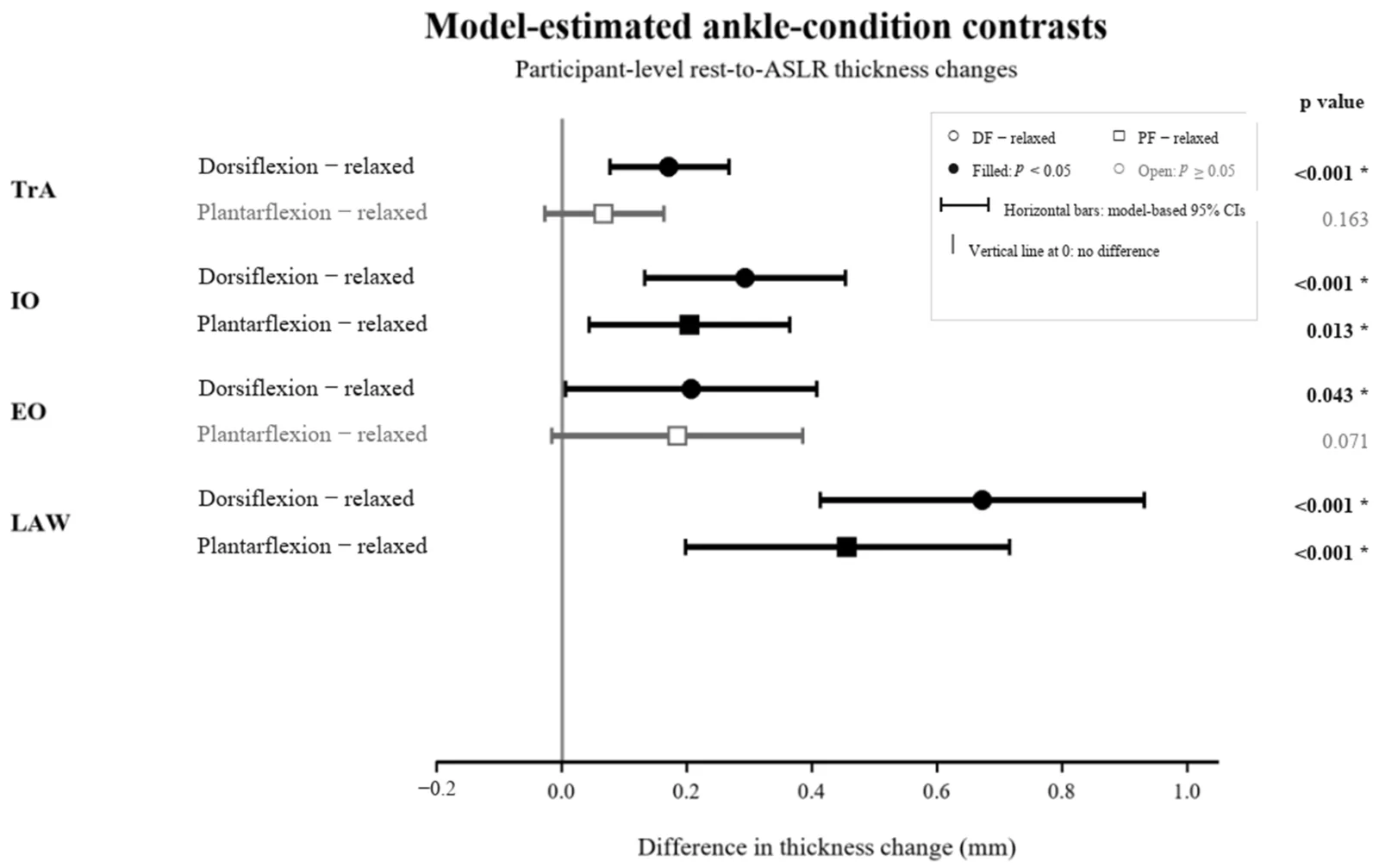
Linear mixed-effects model estimates for changes in participant-level ultrasound thickness. Data points represent planned contrasts comparing the active conditions with the relaxed-ankle reference. Circles and squares indicate the DF-relaxed and PF-relaxed contrasts, respectively. Horizontal bars indicate model-based 95%CIs, and the vertical gray line at zero indicates no difference from the relaxed condition. Black filled symbols and asterisks denote statistically significant contrasts (*p* < 0.05), whereas gray open symbols and lines denote non-significant contrasts. LAW (lateral abdominal wall).

In the exploratory secondary models, the DF condition was associated with significantly greater ΔIO, ΔEO, and change in total lateral abdominal wall thickness compared with the relaxed-ankle condition. The PF condition was associated with greater ΔIO and total lateral abdominal wall thickness changes, whereas the contrast for ΔEO was not statistically significant. Neither active ankle condition significantly altered the relative contribution of TrA (Table 3; Figure 3). The paired sensitivity analyses showed a similar pattern and are reported in Supplementary Table S1.

### 3.3. Pressure Biofeedback Responses

**Figure 4:**
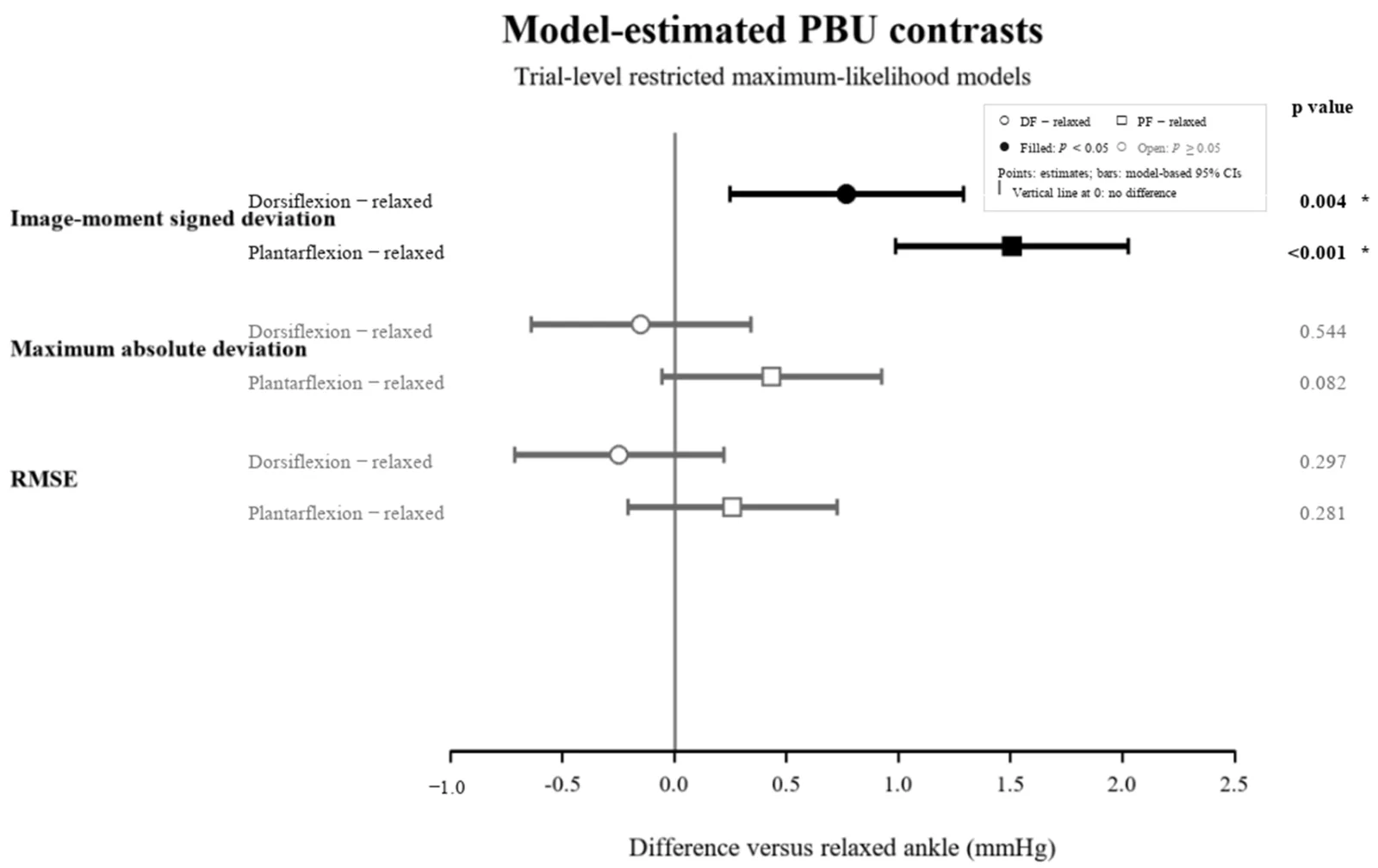
Trial-level restricted maximum likelihood (REML) estimates for PBU outcomes. Points represent the planned contrasts of DF and PF versus the relaxed-ankle reference; circles denote DF, and squares denote PF. Horizontal error bars indicate 95% CIs, and the vertical line at zero indicates no difference from the relaxed-ankle reference. Filled black symbols and asterisks denote statistically significant contrasts (*p* < 0.05), whereas open gray symbols denote nonsignificant contrasts. RMSE, root-mean-square error; mmHg, millimeters of mercury.

PBU responses showed a different pattern from the ultrasound thickness measurements. The mean signed pressure deviation at image acquisition was 2.162 mmHg with a relaxed ankle, 2.929 mmHg with DF, and 3.667 mmHg with PF (Table 2). In the trial-level mixed-effects model, compared with the relaxed-ankle condition, signed pressure deviations significantly increased by 0.768 mmHg under the DF condition (95% CI, 0.248–1.288; *p* = 0.004) and by 1.505 mmHg under the PF condition (95% CI, 0.985–2.025; *p* < 0.001). Neither maximum absolute deviation nor pressure deviation RMSE differed significantly across ankle conditions (Table 4; Figure 4).

### 3.4. Exploratory Ultrasound–PBU Relationship

An exploratory scatterplot revealed no clear linear association between ΔTrA and the signed PBU pressure deviation at the instant of image acquisition. This broad data dispersion supports the premise that ultrasound-derived muscle thickness and lumbopelvic pressure responses should be treated as distinct, complementary outcomes. Supplementary Figure S1 illustrates the distribution of participant-condition values, while Supplementary Table S2 details the intra-rater reliability statistics.

## 4. Discussion

This study addressed a practical reproducibility problem: distal ankle-position cues were associated with differences in selected abdominal and pressure outcomes during a fixed-angle ASLR. The DF condition was associated with greater ΔTrA, ΔIO, and ΔEO values and a greater change in total lateral abdominal wall thickness compared with the relaxed-ankle condition, whereas the PF condition was associated with the largest signed pressure deviation. These findings extend previous ASLR research regarding pelvic pain, abdominal muscle behavior, and lower-limb loading [1,2,3,9,10], aligning with evidence that DF alters tibial nerve excursion and posterior-chain mechanics during straight-leg raising [16,17]. Recent studies also link ASLR performance with abdominal activity and pelvic motion [8] and demonstrate population-related differences in abdominal muscle thickness [19]. Because multiple muscles exhibited morphological changes and ultrasound-derived thickness is not a direct surrogate for electromyographic amplitude, these findings should be interpreted as coordinated task responses rather than selective TrA activation [11,14,15].

The pressure findings provide a complementary measurement perspective. The PF condition significantly increased the signed image-moment pressure deviation, whereas maximum absolute deviation and RMSE did not differ significantly across conditions. This divergence is plausible because the PBU captured discrete pressure events rather than continuous pelvic kinematics, and PBU output is not a direct measure of pelvic neutrality or global trunk stability [4,5,6,7]. Accordingly, ultrasound thickness and PBU pressure should not be treated as interchangeable indicators of a single biomechanical construct.

An additional exploratory observation concerns the abdominal-muscle pattern during PF. Compared with the relaxed-ankle condition, PF was associated with significantly greater ΔIO, whereas the contrast for ΔEO was not statistically significant. Because a formal muscle-by-condition interaction was not tested, this pattern does not establish a definitive between-muscle difference. Although distal ankle positioning may alter force transmission and neuromuscular coordination along myofascial pathways, the Spiral Line remains a conceptual model with mixed empirical evidence regarding anatomical continuity and force transmission [20]. Without concurrent measures of fascial strain, electromyography, ankle torque, contralateral muscle behavior, or three-dimensional pelvic motion, this interpretation supports hypothesis generation rather than mechanistic validation.

Testing sequence warrants particular consideration. Although the three ankle conditions were administered according to a pre-specified schedule containing all six possible condition sequences, all six sequences remained represented after exclusions. The primary model did not include sequence or period as fixed effects. Consequently, potential confounding from fatigue, familiarization, or carryover effects cannot be definitively excluded. Future investigations should prespecify and archive the randomization algorithm and seed, incorporate period and sequence effects into the statistical model (or adopt a formal crossover design framework), and report explicit tests for carryover.

Importantly, these findings do not suggest that any single ankle position is clinically superior. Rather, they demonstrate that distal ankle instructions modify the neuromuscular task demand and introduce systematic variation into ASLR measurements. The primary practical implication is procedural: clinicians and researchers must standardize ankle instructions, image-acquisition timing, and image-aggregation protocols across repeated assessments and report these parameters explicitly.

Several limitations of this study should be noted. First, the sample was restricted to healthy young adults, limiting the generalizability of the findings to clinical populations; the available records did not preserve the recruitment route or a detailed diagnostic screening checklist; and the study was not powered for sex-stratified comparisons. Second, although all six sequences were represented, sequence and period were omitted from the primary analytical models; thus, residual fatigue, familiarization, or carryover effects cannot be completely ruled out. Third, the standardized task was restricted to the dominant lower limb at 30° of hip flexion, and active cues inherently coupled joint position with voluntary muscle contraction. Electromyography, three-dimensional kinematics, ankle joint torque, and perceived exertion were not assessed. Fourth, seven participants were excluded because of poor ultrasound image quality. Finally, PBU readings were recorded manually at discrete events rather than continuously, and the intra-rater MDC_95_ was derived from single-image retests, whereas the primary study outcomes were based on averages of three images. Future studies should expand recruitment to symptomatic cohorts, retain complete sequence-generation metadata, formally model period and sequence effects, assess carryover, and integrate continuous pressure acquisition with complementary electrophysiological and kinematic modalities.

## 5. Conclusions

In healthy young adults, ankle-position instructions influenced selected ultrasound and PBU outcomes during a standardized ASLR. The DF condition was associated with greater changes in TrA thickness and total lateral abdominal wall thickness, whereas the PF condition produced the largest signed PBU pressure deviation at the instant of image acquisition. Although the ΔTrA contrast was statistically significant at the group level, the estimate was smaller than the single-image intra-rater MDC_95_; this comparison remains purely descriptive, as the two statistics address different levels of inference. Standardizing and explicitly reporting ankle positioning and ultrasound image aggregation procedures may improve the consistency and interpretability of ASLR-based rehabilitation assessments. These findings warrant confirmation in symptomatic clinical populations before being used to guide patient-level clinical decision-making.

## Figures and Tables

**Table 1:** Participant characteristics and ankle-angle manipulation.

Variable	Mean or Count	SD
Age, years	24.70	1.74
Height, cm	169.30	7.38
Weight, kg	63.45	10.96
BMI, kg/m^2^	22.03	2.70
Sex, male/female	17/16	NA
Dominant leg, right	33	NA
Relaxed ankle position, reference angle, degrees	0.00	NA
Maximal DF excursion from relaxed baseline, degrees	31.84	6.31
Maximal PF excursion from relaxed baseline, degrees	25.42	6.82

BMI, body mass index; DF, dorsiflexion; NA, not applicable; PF, plantarflexion; SD, standard deviation.

**Table 2:** Absolute and change-score outcomes by ankle condition, presented as mean (SD).

Outcome	Relaxed	DF	PF
TrA thickness at rest, mm	2.431 (0.608)	2.472 (0.632)	2.415 (0.538)
TrA thickness during ASLR, mm	2.588 (0.670)	2.776 (0.759)	2.627 (0.686)
ΔTrA, mm	0.144 (0.324)	0.316 (0.406)	0.212 (0.339)
ΔIO, mm	−0.009 (0.703)	0.284 (0.820)	0.195 (0.686)
ΔEO, mm	−0.388 (0.680)	−0.180 (0.749)	−0.203 (0.639)
Lateral abdominal wall change, mm	−0.253 (1.049)	0.420 (1.308)	0.204 (0.981)
Relative TrA during ASLR	0.202 (0.034)	0.209 (0.039)	0.204 (0.041)
PBU image-moment deviation, mmHg	2.162 (3.971)	2.929 (3.357)	3.667 (3.153)
PBU maximum absolute deviation, mmHg	4.061 (2.872)	3.909 (2.797)	4.495 (2.291)
PBU RMSE, mmHg	3.445 (3.061)	3.197 (2.995)	3.702 (2.602)

Note: Absolute TrA thickness values represent participant-condition means in millimeters. The change in total lateral abdominal wall thickness was calculated using unrounded participant-level data. Consequently, the sum of the displayed individual muscle components may differ slightly from the displayed total due to rounding.

**Table 3:** Mixed-effects model estimates for participant-level ultrasound thickness changes.

Outcome	Model Term or Contrast	Estimate	95% CI	*p*
ΔTrA	DF—relaxed	0.172	0.077 to 0.267	<0.001
ΔTrA	PF—relaxed	0.068	−0.027 to 0.163	0.163
ΔIO	DF—relaxed	0.293	0.133 to 0.454	<0.001
ΔIO	PF—relaxed	0.204	0.044 to 0.365	0.013
ΔEO	DF—relaxed	0.207	0.007 to 0.408	0.043
ΔEO	PF—relaxed	0.185	−0.016 to 0.386	0.071
Lateral abdominal wall change	DF—relaxed	0.672	0.413 to 0.931	<0.001
Lateral abdominal wall change	PF—relaxed	0.457	0.198 to 0.716	<0.001
Relative TrA contribution	DF—relaxed	0.004	−0.004 to 0.011	0.322
Relative TrA contribution	PF—relaxed	0.000	−0.008 to 0.007	0.913

Note: Estimates represent planned contrasts comparing the active conditions against the relaxed-ankle reference, derived from linear mixed-effects models of 99 participant-condition change values (ASLR minus rest). Each state-specific value represents the average of three end-expiratory image measurements.

**Table 4:** Trial-level mixed-effects estimates for PBU outcomes.

Outcome	Contrast	Estimate	95% CI	*p*
Image-moment signed deviation	DF—relaxed	0.768	0.248 to 1.288	0.004
Image-moment signed deviation	PF—relaxed	1.505	0.985 to 2.025	<0.001
Maximum absolute deviation	DF—relaxed	−0.152	−0.642 to 0.338	0.544
Maximum absolute deviation	PF—relaxed	0.434	−0.056 to 0.924	0.082
RMSE	DF—relaxed	−0.248	−0.715 to 0.218	0.297
RMSE	PF—relaxed	0.257	−0.210 to 0.723	0.281

## Data Availability

The data presented in this study are available from the corresponding author upon reasonable request. The data are not publicly available because of institutional ethics and participant-consent restrictions. Generative artificial intelligence tools were used only to assist with translation and language editing. The authors reviewed and verified the manuscript and take full responsibility for its content.

## Supplementary Materials

The following supporting information can be downloaded at: https://www.mdpi.com/article/10.3390/jfmk11030337/s1, Table S1: Participant-level paired sensitivity analyses for selected ultrasound outcomes; Figure S1: Exploratory association between participant-condition mean TrA thickness change and participant-condition mean PBU signed deviation at image acquisition; Table S2: Within-examiner reliability of single-image means rebuilt from three measurements.
