# Supplementary material for: Standardizing Ankle Position During the Active Straight Leg Raise: Effects on Lateral Abdominal Muscle Thickness and Pressure Biofeedback in Healthy Young Adults
Source: J Funct Morphol Kinesiol. 2026 Aug 27;11(3):337. doi: 10.3390/jfmk11030337 (PMC13608828; doi:10.3390/jfmk11030337)
Supplement: Supplementary file 1 [file jfmk-11-00337-s001.zip › jfmk-4465866-supplementary.pdf]

Supplementary Materials

Supplementary Table S1. Participant-level paired sensitivity analyses for selected ultrasound outcomes.

Outcome	Contrast	Estimate	95% CI	Holm p	dz
TrA change	Dorsiflexion - relaxed	0.172	0.075 to 0.268	0.002	0.631
TrA change	Plantarflexion - relaxed	0.068	-0.041 to 0.176	0.212	0.222
IO change	Dorsiflexion - relaxed	0.293	0.094 to 0.492	0.010	0.523
IO change	Plantarflexion - relaxed	0.204	0.037 to 0.372	0.018	0.433
Lateral abdominal wall change	Dorsiflexion - relaxed	0.672	0.381 to 0.964	<0.001	0.817
Lateral abdominal wall change	Plantarflexion - relaxed	0.457	0.190 to 0.724	0.001	0.606
Relative TrA change	Dorsiflexion - relaxed	0.004	-0.003 to 0.010	0.562	0.191
Relative TrA change	Plantarflexion - relaxed	0.000	-0.009 to 0.008	0.926	-0.016

Abbreviation: dz (paired-samples standardized mean difference).

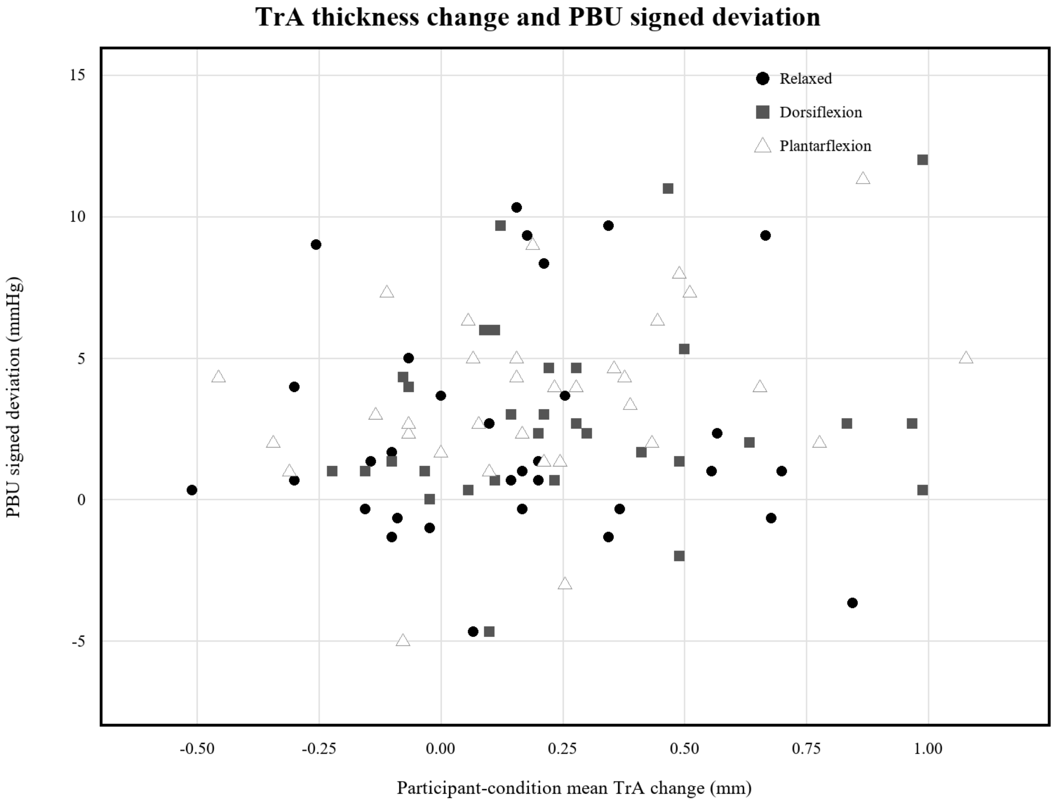

Supplementary Figure S1. Exploratory association between participant-condition mean TrA thickness change and participant-condition mean PBU signed deviation at image acquisition. PBU means were calculated directly from the

three trial records for each condition. Each ultrasound change was based on three end-expiratory images at rest and three during ASLR. Marker shape and grayscale identify ankle condition; no pooled regression line is shown because repeated observations within participants are not independent. Abbreviations: ASLR (active straight leg raise); PBU (pressure biofeedback unit); TrA (transversus abdominis).

Supplementary Table S2. Within-examiner reliability of single-image means rebuilt from three measurements.

Muscle	Images, n	ICC(A,1)	Bootstrap 95% CI	SEM, mm	MDC95, mm	Mean absolute difference, mm
TrA	50	0.847	0.714 to 0.929	0.172	0.477	0.167
IO	50	0.893	0.689 to 0.973	0.411	1.139	0.350
EO	50	0.848	0.717 to 0.934	0.396	1.098	0.357
